# Distinctive Cytokines as Biomarkers Predicting Fatal Outcome of Severe *Staphylococcus aureus* Bacteremia in Mice

**DOI:** 10.1371/journal.pone.0059107

**Published:** 2013-03-08

**Authors:** Sanne van den Berg, Jon D. Laman, Louis Boon, Marian T. ten Kate, Gerjo J. de Knegt, Rob M. Verdijk, Henri A. Verbrugh, Jan L. Nouwen, Irma A. J. M. Bakker-Woudenberg

**Affiliations:** 1 Department of Medical Microbiology and Infectious Diseases, Erasmus University Medical Centre, Rotterdam, The Netherlands; 2 Department of Immunology, Erasmus University Medical Centre, Rotterdam, The Netherlands; 3 Bioceros BV, Utrecht, The Netherlands; 4 Department of Pathology, Erasmus University Medical Centre, Rotterdam, The Netherlands; Louisiana State University Health Sciences Center, United States Of Ameica

## Abstract

Invasive *Staphylococcus aureus* infections are frequently associated with bacteraemia. To support clinical decisions on antibiotic therapy, there is an urgent need for reliable markers as predictors of infection outcome. In the present study in mice, bacteraemia was established by intravenous inoculation of a clinical *S. aureus* isolate at the LD_50_ inoculum. As potential biomarkers for fatal outcome, blood culture (qualitative and quantitative), serum levels of C-reactive protein (CRP), as well as 31 selected cytokines and chemokines were assessed during the first three days of infection. A positive *S. aureus* blood culture, the quantitative blood culture, CRP levels, and levels of eight cytokines were indicative for the presence of *S. aureus* bacteraemia. However, only tumor necrosis factor (TNF) α, interleukin (IL) 1α, and keratinocyte chemoattractant (KC; a functional homologue of human IL-8) were each significantly elevated in eventually non-surviving infected mice versus eventually surviving infected mice. In severe *S. aureus* bacteraemia in mice, TNF-α, IL-1α, and KC are biomarkers predicting fatal outcome of infection. KC was a biomarker elevated irrespective the progression of infection, which is very interesting regarding clinical application in view of the heterogeneity of patients experiencing bacteraemia in this respect.

## Introduction

*Staphylococcus aureus* is an important opportunistic pathogen that causes a variety of infections, from relatively mild infections such as skin infections and food poisoning, to life-threatening conditions such as necrotizing pneumonia and osteomyelitis [1]. The invasive infections are frequently associated with *S. aureus* bacteremia [2]. Shorr *et al.* showed that *S. aureus* was the most common bacterial isolate in 6,697 bloodstream infections in the USA, and *S. aureus* was more strongly associated with mortality than any other bacterial pathogen [3].

A clinically significant bacteremia is generally defined as the isolation of bacteria from one or more peripheral venous blood culture samples collected from a patient with associated relevant clinical symptoms of systemic infection as defined on the 2001 International Sepsis Definitions Conference [4]. When sepsis is strongly suspected, an appropriate treatment regimen for each patient is designed.

Blood culture confirms the presence of bacteremia, and allows identification of the causative infectious agent. Rapid diagnosis and (antibiotic) treatment of bacteremia is essential, as any delay in treatment may lead to worse outcome. On the other hand, antibiotic use should be limited, and proper decision on initiating antibiotic therapy or adjustment of antibiotic choice in therapy already started in severe bacteremia is highly important. Knowledge of how to predict fatal outcome in patients with *S. aureus* bacteremia is currently lacking. To support clinical decisions on these issues, there is an urgent need for reliable markers that can be used as predictors of outcome of infection.

An obvious candidate biomarker may be C-reactive protein (CRP), which is the classic acute phase protein. CRP is released by the liver to the blood, and activates the complement system. This marker is currently used to determine inflammation and tissue damage [5]. Systemic levels of CRP are elevated in septic traumatized patients compared to non-septic traumatized patients [6]. Synthesis of CRP by hepatocytes starts very rapidly, and CRP can be detected in plasma after 6–12 hours, and a plateau is reached after 20–72 hours. Plasma half-life of CRP is about 19 hours [7]–[9].

Bacteremia can lead to sepsis and multiple organ failure, and eventually to death when therapy fails. For many years, it was assumed that sepsis is a consequence of an overwhelming inflammatory reaction of the patient to microorganisms. Neutralization of single pro-inflammatory cytokines like tumor necrosis factor (TNF) α or interleukin (IL) 1 in animal models of sepsis resulted in protection against lethal sepsis [10]–[14]. In contrast to these murine studies, inhibition of these cytokines has not provided clinical benefit to patients with severe sepsis [15]–[20]. Therefore, it is clear that not only the pro-inflammatory reaction forms the basis for adverse outcome in sepsis, immune suppression by anti-inflammatory reactions is involved as well. As cytokines are important in sepsis, they are candidate biomarkers for presence and fatal outcome of *S. aureus* bacteremia. Clinical studies determined cytokine levels in sepsis or sepsis-like situations. Two studies assessed cytokine levels in healthy volunteers after lipopolysaccharide injection (LPS) injection. Dandona *et al.* [21] found elevated serum levels of TNF-α, and Van der Poll *et al.* [22] described a rise in IL-10 plasma levels. Cytokine levels in septic patients were measured as well. These studies did not focus on a single causative infectious agent. Levels of IL-1β [23], IL-2 [24], IL-6 [23], [25]-[28], IL-8 [24], IL-10 [22], [23], [26]-[28], TNF-α [23], [28], [29], and IFN-γ [28] were elevated in septic patients compared to normal levels. Levels of IL-6 [23], [26], IL-10 [22], [30] and TNF-α [30] were predictive for fatal outcome in patients with sepsis, while IL-10 [23], [26] levels were predictive for survival. It is difficult to evaluate the value of changes in cytokine profile during bacteremia in patients due to differences in the causative infectious agents in these studies. In addition, only a limited number of cytokines were included in these studies, which hinders the comparison of results obtained from the various studies.

Hence, we hypothesized that cytokines are useful as biomarkers for fatal outcome of *S. aureus* bacteremia. The present study in mice was performed to identify biomarkers predicting fatal outcome of severe *S. aureus* bacteremia. We focused on blood culture, CRP, and selected cytokines. To this aim, we established an *in vivo* model of severe *S. aureus* bacteremia in mice, using a clinical *S. aureus* isolate. The use of a *S. aureus* inoculum resulting in 50% mortality allowed comparing eventually surviving infected mice and eventually non-surviving infected mice in the same model.

## Materials and Methods

### Bacteria

A clinical *S. aureus* isolate (isolate P), recovered from a septic patient, was used. This isolate was kindly supplied by G. Buist (University Medical Centre Groningen, The Netherlands) and described by Ziebandt *et al.* (CA-MSSA, MLST type 7, *agr*-type 1, *pvl*-negative) [31]. Staphylococci were grown overnight at 35°C on Colombia III blood agar (Becton Dickinson, Breda, The Netherlands). Cultures of *S. aureus*, grown in Brain Heart Infusion broth (Becton Dickinson, Breda, The Netherlands) until OD_560_ ∼ 1.0, were stored at –80°C.

### Animals

Specified opportunistic pathogen-free (SOPF) female BALB/cBYJ *S. aureus*-free mice (11-13 weeks at day of infection) were obtained from Charles River (Saint-Germain-sur-l’Arbresle, France), and were given food and water *ad libitum*. Before each experiment, *S. aureus*-free status was checked by culture of fresh fecal and nasal microbiota as well as by confirming the absence of anti-staphylococcal IgG levels against 54 antigens using Luminex technology [32].

### Ethics statement

The experimental protocols adhered to the rules specified in the Dutch Animal Experimentation Act (1977) and the published Guidelines on the Protection of Experimental Animals by the Council of the EC (1986). The Institutional Animal Care and Use Committee of the Erasmus University Medical Centre Rotterdam approved the present protocols.

### *S. aureus* bacteremia

A suspension of *S. aureus* was defrosted and centrifuged for 10 minutes at 14.000×*g. *. The *S. aureus* pellet was resuspended in saline, and diluted to obtain the desired inoculum. To establish bacteremia, 100 µL of *S. aureus* isolate P was injected into the tail vein.

Various *S. aureus* inocula, resulting in 0 – 100% mortality, were injected intravenously (n = 5 per inoculum) to determine the inoculum-dependent cumulative mouse mortality. Clinical signs of illness in each mouse were evaluated twice daily. Mice with bad fur were scored -2. Mice with bad fur and hunched back were scored -3. Mice with bad fur and hunched back and that were instable, were scored -4. These mice showed severe signs of illness and were euthanized by CO_2_ exposure. Euthanized mice were considered as death, as pilot experiments showed that mice with severe signs of illness died before the end of the experiment. Animal survival 14 days after inoculation was monitored and cumulative mortality was calculated. For experiments, groups of mice (n = 25) were infected with a *S. aureus* inoculum at the 50% lethal dose (LD_50_). Animal survival rate, body weight, and discomfort were monitored over 14 days. From mice that were euthanized due to severe signs of illness, blood, lung, spleen, liver, and kidneys were cultured to confirm that they died because of *S. aureus* infection only.

At various intervals after infection, the bacterial load in blood and infected organs was determined. Mice (n = 4 per time point) were sacrificed at 1, 17, or 48 hours by CO_2_ exposure. A blood sample was taken via (transcutaneous) cardiac puncture and collected in a vial containing Lithium Heparin (Sarstedt, Etten-Leur, The Netherlands). The lung, spleen, liver, and kidneys were removed aseptically and homogenized (Polytron, Kinematica, Luzern, Switzerland) in 2 mL of saline for 10 seconds at 30,000 rpm at room temperature. Undiluted homogenate suspensions and blood and 10-fold serial dilutions of homogenates and blood in saline were plated onto Colombia III blood agar. After overnight incubation at 35°C colonies were counted.

The histopathological changes in infected tissues of non-surviving, euthanized mice (n = 4) were determined in animals sacrificed using an overdose of pentobarbital (Ceva Sante Animale, Naaldwijk, The Netherlands). The *in situ* re-expanded lungs and the other organs were processed as described before [33]. Tissue sections (4 µm) were stained with haematoxylin and eosin. A pathologist examined the sections microscopically.

### Parameters to monitor infection progression and outcome

#### Blood cultures

Blood was withdrawn from the tail artery of infected mice at 17, 48, and 72 hours after infection (n = 25), collected in a Microvette® CB300 capillary tube containing Lithium Heparin (Sarstedt, Etten-Leur, The Netherlands) and cultured as described above.

#### C-reactive protein

For quantification of CRP, blood was withdrawn from the tail artery of infected mice at 1, 17, or 48 hours after infection. Blood was collected in a Microvette^®^ CB300 capillary tube (Sarstedt, Etten-Leur, The Netherlands) and sera were prepared and stored at –80°C. To exclude effects of animal handling, a control group receiving saline only was included (n = 10). CRP was measured via solid phase ELISA kit (LifeDiagnostics, West Chester, PA) according to the manufacturer’s instructions, as described by Reichelt *et al.* [34]. All samples were run in duplicate and reported as an average of two determinations.

#### Cytokines

Cytokine concentrations in serum were assessed at 1, 17, or 48 hours after infection. To exclude effects of animal handling on cytokine levels, a control group receiving saline only was included (n = 10). Cytokine levels were determined using the Milliplex^®^ MAP kit (Millipore, Amsterdam, The Netherlands) in duplicate, following the manufacturer’s user manual using the Luminex 100 instrument (Biomedical Diagnostics, Antwerpen, Belgium). Selected cytokines were: granulocyte colony stimulating factor (G-CSF), granulocyte monocyte colony stimulating factor (GM-CSF), macrophage colony stimulating factor (M-CSF), interferon (IFN) γ, leukemia inhibitory factor (LIF), tumor necrosis factor (TNF) α, interleukin (IL) 1α, IL-1β, IL-2, IL-3, IL-4, IL-5, IL-6, IL-7, IL-10, IL-12p40, IL-12p70, IL-13, IL-15, and IL-17A. Selected chemokines were: eotaxin (CCL1), IFN-γ inducible protein of 10 kDa (IP-10; CXCL10), keratinocyte chemoattractant (KC; CXCL1; functional homologue of human IL-8 [35]), lipopolysaccharide induced CXC chemokine (LIX; CXCL5-6), monocyte chemoattractant protein 1 (MCP-1; CCL2), monokine induced by IFN-γ (MIG; CXCL9), macrophage inflammatory protein 1α (MIP-1α; CCL3), MIP-1β (CCL4), MIP-2 (CXCL2), the chemokine regulated-upon-activation normal T-cell expressed and secreted (RANTES; CCL5), and vascular endothelial growth factor (VEGF). Selected cytokines and chemokines are representative for both pro- and anti-inflammatory cytokines.

#### Statistical analysis

Quade’s rank analysis of covariance was used to compare body weight of placebo-inoculated mice, surviving infected mice and non-surviving infected mice. Fisher exact test was used to compare numbers of mice with positive blood cultures in groups of surviving infected mice versus non-surviving infected mice. Also, *S. aureus* counts in blood and organs, and serum levels of CRP and cytokines in groups of placebo-inoculated mice, surviving infected mice and non-surviving infected mice were compared using the Mann-Whitney *U* test. Binary logistic regression analysis using all variables associated with presence or fatal outcome of *S. aureus* bacteremia in the Mann-Whitney *U* test (*P*-values < 0.01) was performed to determine the biomarkers for presence and fatal outcome of *S. aureus* bacteremia. Receiver operating characteristic (ROC) curves were constructed and markers with area under ROC curve > 0.8 are considered indicative as biomarker.

## Results

### Course of *S. aureus* bacteremia

**Figure 1:**
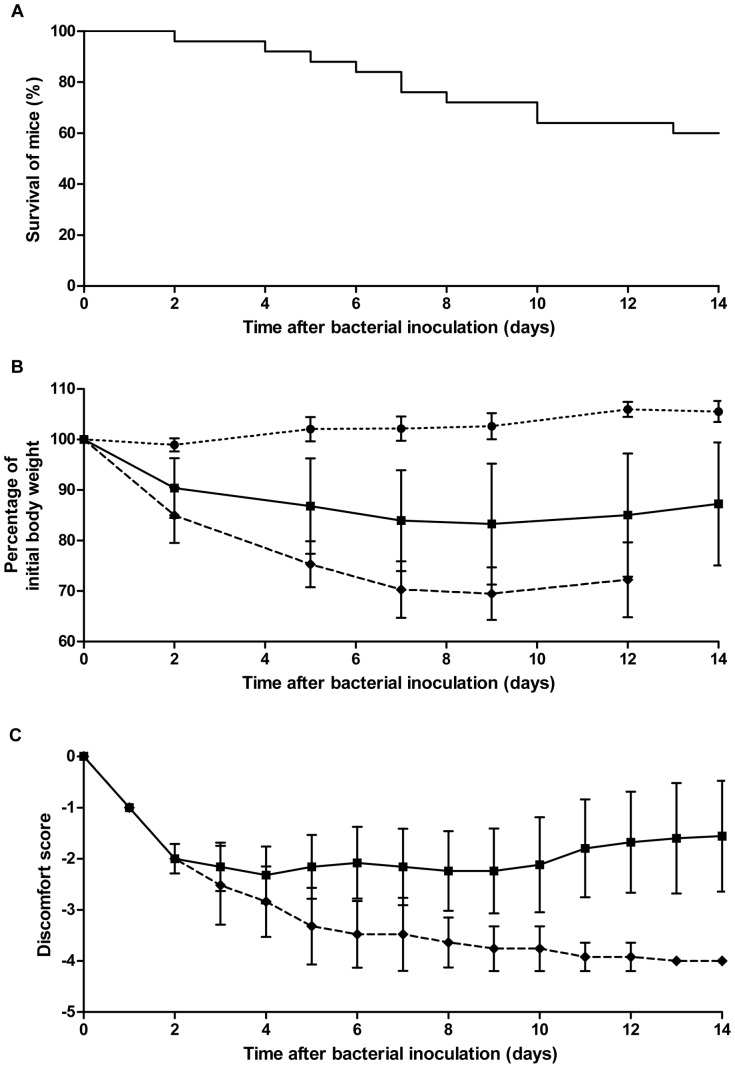
Time course of bacteremia in mice infected with *S. aureus* at the LD_50_ inoculum. **A** Survival of infected mice (n = 25). **B** Mouse body weight, shown as mean ± SD (error bars). Body weight of non-surviving infected mice was significantly lower compared to surviving infected mice and to placebo-inoculated mice (*P* < 0.01; Quade’s rank analysis of covariance). **C** Discomfort score, shown as mean ± SD (error bars). Discomfort score -2: bad fur. Discomfort score -3: bad fur, hunched back. Discomfort score -4: bad fur, hunched back, instability, euthanasia needed. Placebo-inoculated mice (• n = 12), surviving infected mice (▪ n = 25), non-surviving infected mice (♦n = 25).

**Figure 2:**
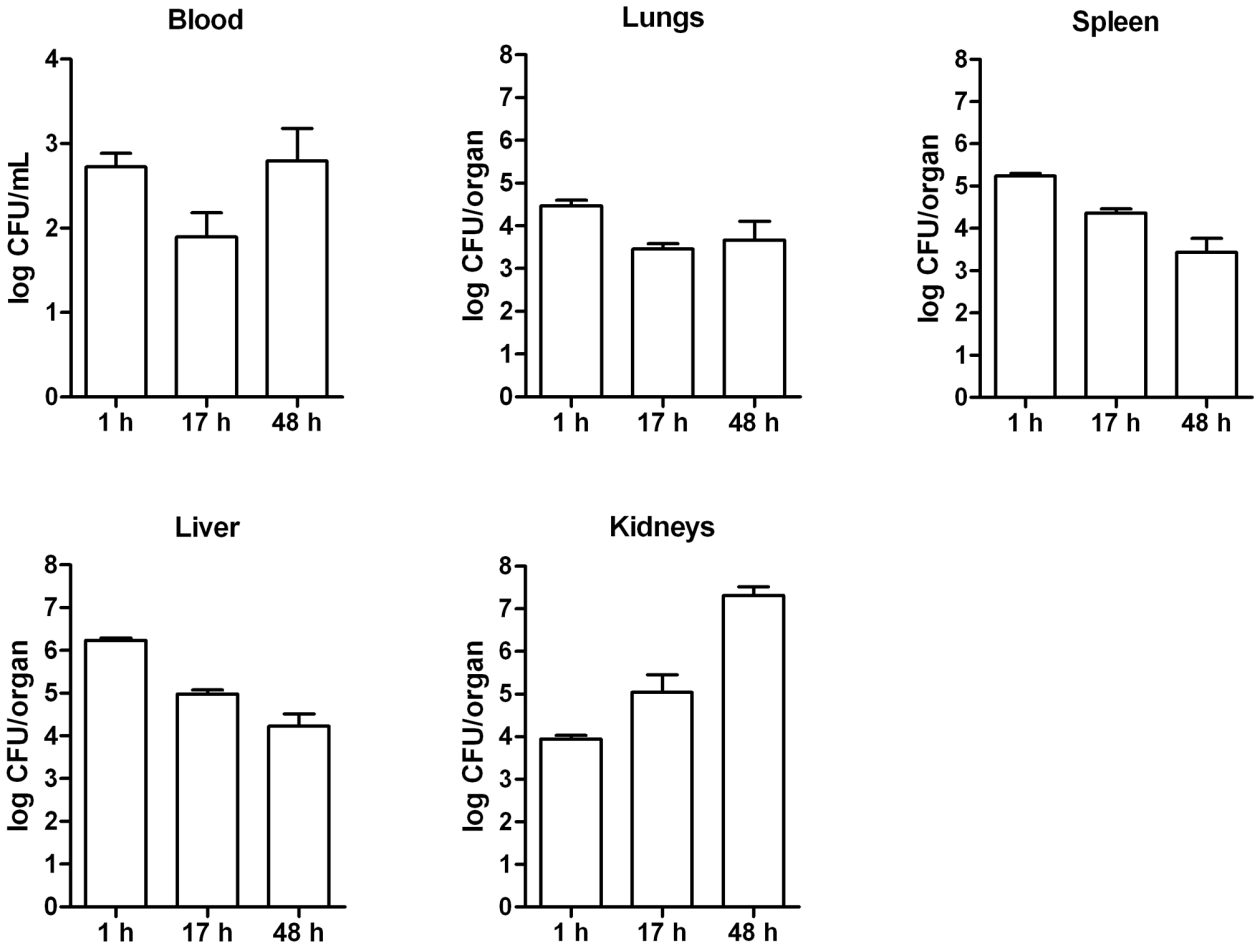
*S. aureus* counts in blood and organs from mice with bacteremia. At indicated time points after intravenous inoculation of *S. aureus* at the LD_50_ inoculum, mice were sacrificed and quantitative cultures of blood and organs were performed (n = 4 per time point). Results are expressed as mean ± SD (error bars). Statistically significant differences (Mann-Whitney *U* test) in *S. aureus* load were not found. h, hours.

To establish the LD_50_ inoculum in the *S. aureus* bacteremia model, groups of mice were intravenously infected with different inocula (data not shown). *S. aureus* at 1 × 10^4^ CFU did not result in mortality, while 2 × 10^6^ CFU *S. aureus* resulted in 100% mortality. The LD_50_ inoculum was calculated to be 3 × 10^5^ CFU. The survival of mice infected with the LD_50_ inoculum declined gradually over 14 days (Fig. 1*A*). After this time point, no changes in animal survival were observed. Infected mice decreased in body weight compared to placebo-inoculated mice. Body weight of eventually non-surviving infected mice was significantly lower compared to eventually surviving infected mice and to placebo-inoculated mice (*P* < 0.01; Quade’s rank analysis of covariance; Fig. 1*B*). Discomfort increased in all infected mice from two days after infection. In surviving infected mice, this remained stable until day 10 after infection and then decreased again, whereas the discomfort score in non-surviving infected mice further increased. Animals that showed severe signs of illness were euthanized and were scored –4 (Fig. 1*C*). During the course of bacteremia, *S. aureus* load in blood and organs did not significantly change over time (Fig. 2). Only in kidneys, *S. aureus* load tended to increase over time.

**Figure 3:**
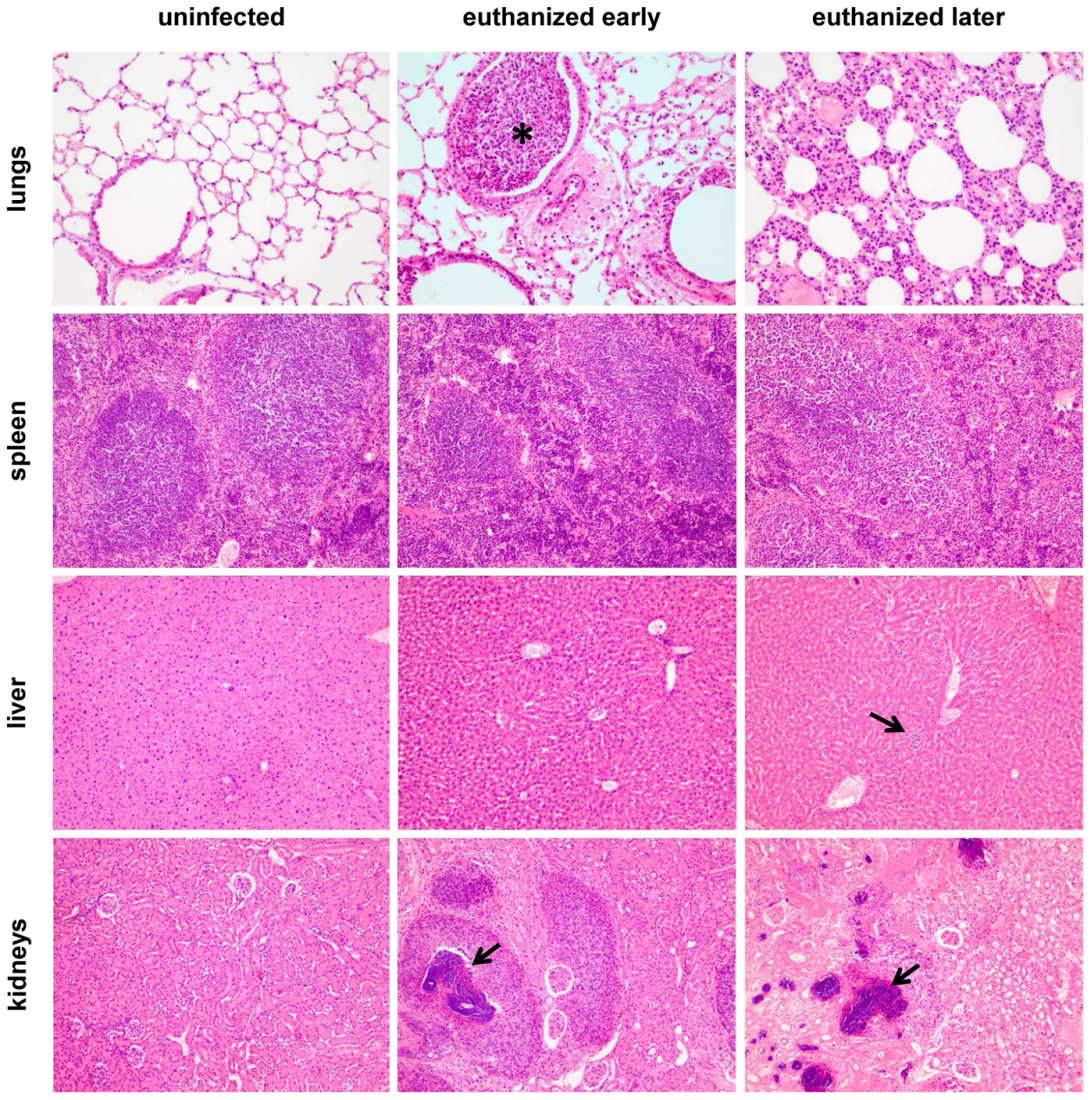
Representative histopathological features in lungs, spleen, liver and kidneys of mice with *S. aureus* bacteremia. Haematoxylin and eosin stained tissue sections are shown. Lungs (200×), spleen (100×), liver (100×), and kidneys (100×). Uninfected mice; mice with *S. aureus* bacteremia that were euthanized early (day 4-5); mice with *S. aureus* bacteremia that were euthanized later (day 10–11). In lungs, asterisk indicates bronchopneumonia with neutrophils. In liver, arrow indicates hepathic microabscesses with neutrophils. In kidneys, arrows indicate abscesses in kidneys.

The course of *S. aureus* bacteremia was further characterized by histopathology of lungs, spleen, liver, and kidneys. Organs of infected mice that had to be euthanized early (day 4-5) and mice that had to be euthanized later (day 10–11) were compared (Fig. 3). Most striking were renal bacterial abscesses in all euthanized mice. Bronchopneumonia was observed in both early euthanized mice and in one of the late euthanized mice. Bronchioles were filled with neutrophils. The hepatic sinusoids were dilated with a subtle increase of sinusoidal neutrophils in mice that had to be euthanized early. Mice that had to be euthanized late also developed hepatic microabscesses. The spleen showed lymphodepletion, which was more profound in late euthanized mice compared to early euthanized mice. *S. aureus* was primarily found intra- and perivascularly.

### *S. aureus* load in blood in surviving infected mice versus non-surviving infected mice

**Figure 4:**
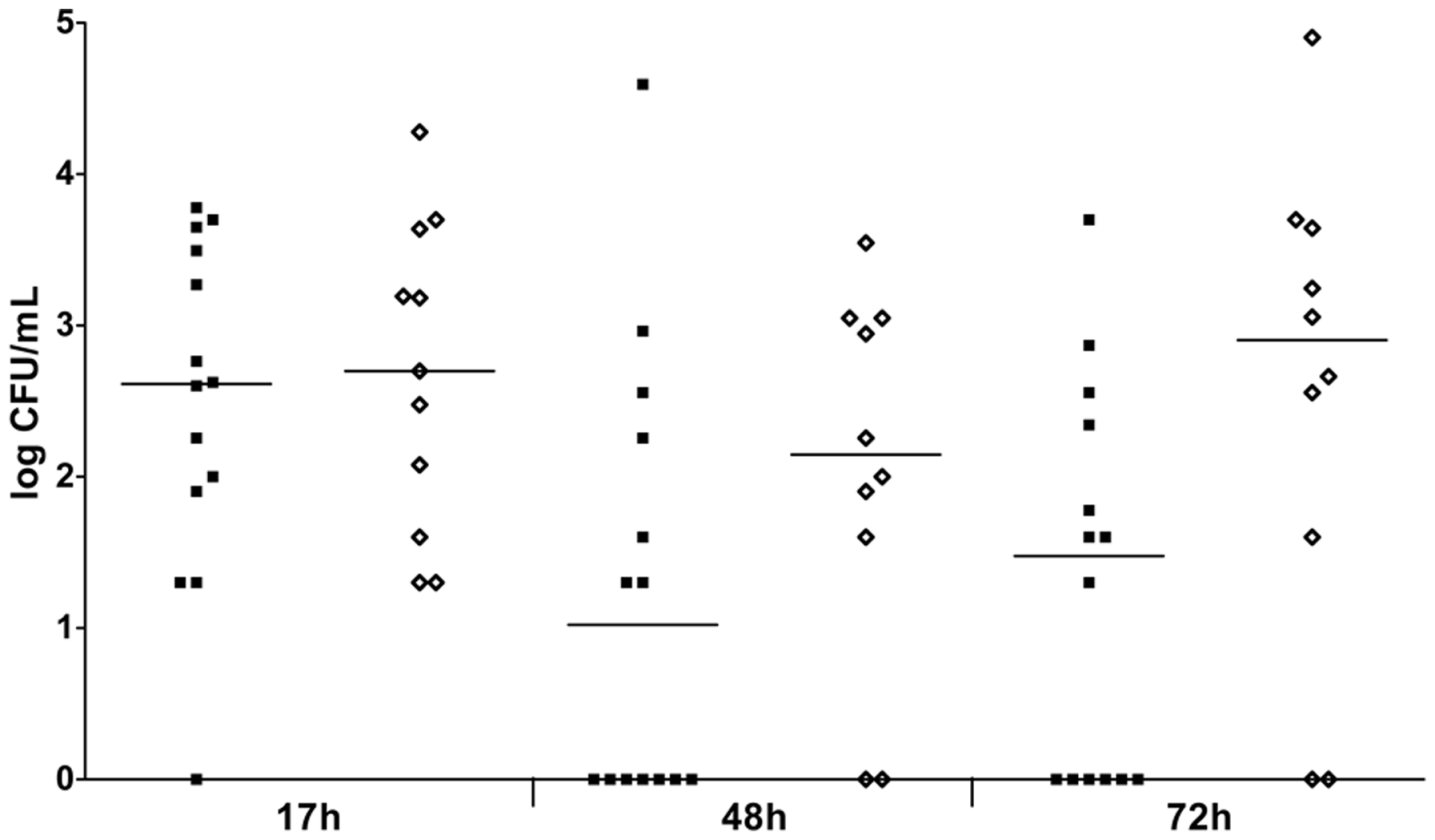
*S. aureus* counts in blood from mice with bacteremia at indicated time points. Mice were inoculated intravenously with *S. aureus* at the LD_50_ inoculum (n = 25). Each symbol represents a single mouse. Median values are indicated by horizontal lines. Surviving infected mice (▪), non-surviving infected mice (⋄). Statistically significant differences in numbers of animals with positive blood cultures (Fisher exact test) or *S. aureus* counts (Mann-Whitney *U* test) between groups of surviving infected mice versus non-surviving infected mice were never observed. h, hours.

We determined whether conventional blood culture is indicative for fatal infection outcome (Fig. 4). The number of animals with positive blood cultures was not significantly different between groups of surviving infected mice and non-surviving infected mice at all time points. Quantitative blood cultures showed substantial inter-individual variability. At 72 hours, CFU counts tended to be higher in non-surviving infected mice compared to surviving infected mice, although this was not statistically significant. Blood cultures were negative in all surviving infected animals at 2 weeks after inoculation (data not shown).

### C-reactive protein levels in surviving infected mice versus non-surviving infected mice

**Figure 5:**
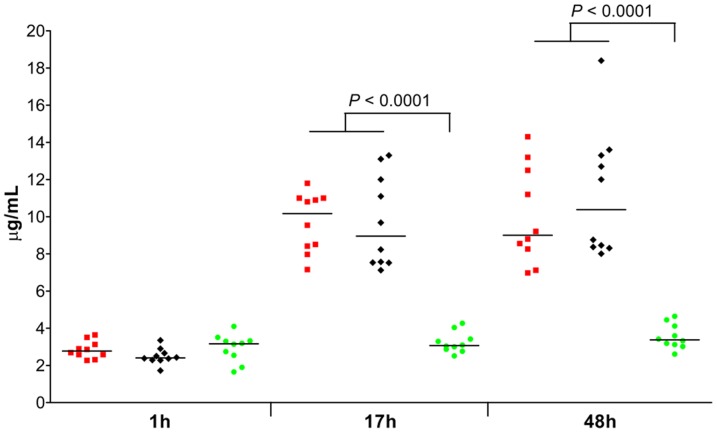
Serum C-reactive protein (CRP) levels in mice with *S. aureus* bacteremia at indicated time points. Mice were inoculated intravenously with *S. aureus* at the LD_50_ inoculum. Each symbol represents a single mouse. Red squares represent surviving infected mice (n = 10); black diamonds represent non-surviving infected mice (n = 10); green circles represent placebo-inoculated mice (n = 10). Median values are presented by horizontal lines. Statistically significant differences between infected mice and placebo-inoculated mice are indicated. Statistically significant differences were never found between surviving infected mice and non-surviving infected mice. *P*-values < 0.01 (Mann-Whitney *U* test) are shown. h, hours.

We determined whether serum CRP levels are indicative for presence and fatal outcome of *S. aureus* bacteremia in mice (Fig. 5). CRP levels were significantly elevated in infected mice compared to placebo-inoculated mice at both 17 and 48 hours after infection (*P* < 0.01). No significant differences were found between surviving infected mice and non-surviving infected mice.

### Cytokines in surviving infected mice versus non-surviving infected mice

Serum levels of 31 selected cytokines were assessed. Significant differences between *S. aureus*-infected mice versus placebo-inoculated mice as well as between non-surviving infected mice versus surviving infected mice are shown in Table 1.

**Table 1:** Cytokines in serum from mice with *S. aureus* bacteremia at various time points after infection.

	Elevated due to infection^a^			Elevated in non-surviving infected mice versus surviving infected mice^a^		
**Cytokine/chemokine**	**1h**	**17h**	**48h**	**1h**	**17h**	**48h**
G-CSF		**<0.0001**	**<0.0001**			
GM-CSF		0.0044				
M-CSF		0.0039	0.0099			
IFN-γ		<0.0001	0.0017			
TNF-α	**<0.0001**	**<0.0001**	**<0.0001**			**0.0004**
IL-1α		**0.0073**	**0.0001**			**0.0065**
IL-1β		0.0037	0.0031			0.0028
IL-6	**<0.0001**	**<0.0001**	**<0.0001**			
IL-10	0.0001	0.0003				
IL-12p70		**<0.0001**	**0.0001**			
IL-15		0.0001	0.0003			
IL-17A		0.0002	0.0007			
eotaxin (CCL1)			0.0012			0.0025
IP-10 (CXCL10)		**<0.0001**	**0.0008**			
KC (CXCL1)	**0.0089**	**<0.0001**	**<0.0001**	**0.0043**	**0.0025**	**0.0090**
MCP-1 (CCL2)		<0.0001	<0.0001			
MIG (CXCL9)		0.0001	0.0008			
MIP-1α (CCL3)		**0.0001**	**0.0019**			
MIP-1β (CCL4)		0.0015				
MIP-2 (CXCL2)		0.0003				
RANTES (CCL5)		0.0024				

a Only *P*-values for the 21 cytokines that were significantly elevated (Mann-Whitney *U* test, *P* < 0.01) in infected mice (n = 20) compared to placebo-inoculated mice (n = 10) are shown. Cytokines for which area under ROC curve was > 0.8 (logistic regression analysis) are indicated in bold.

**Figure 6:**
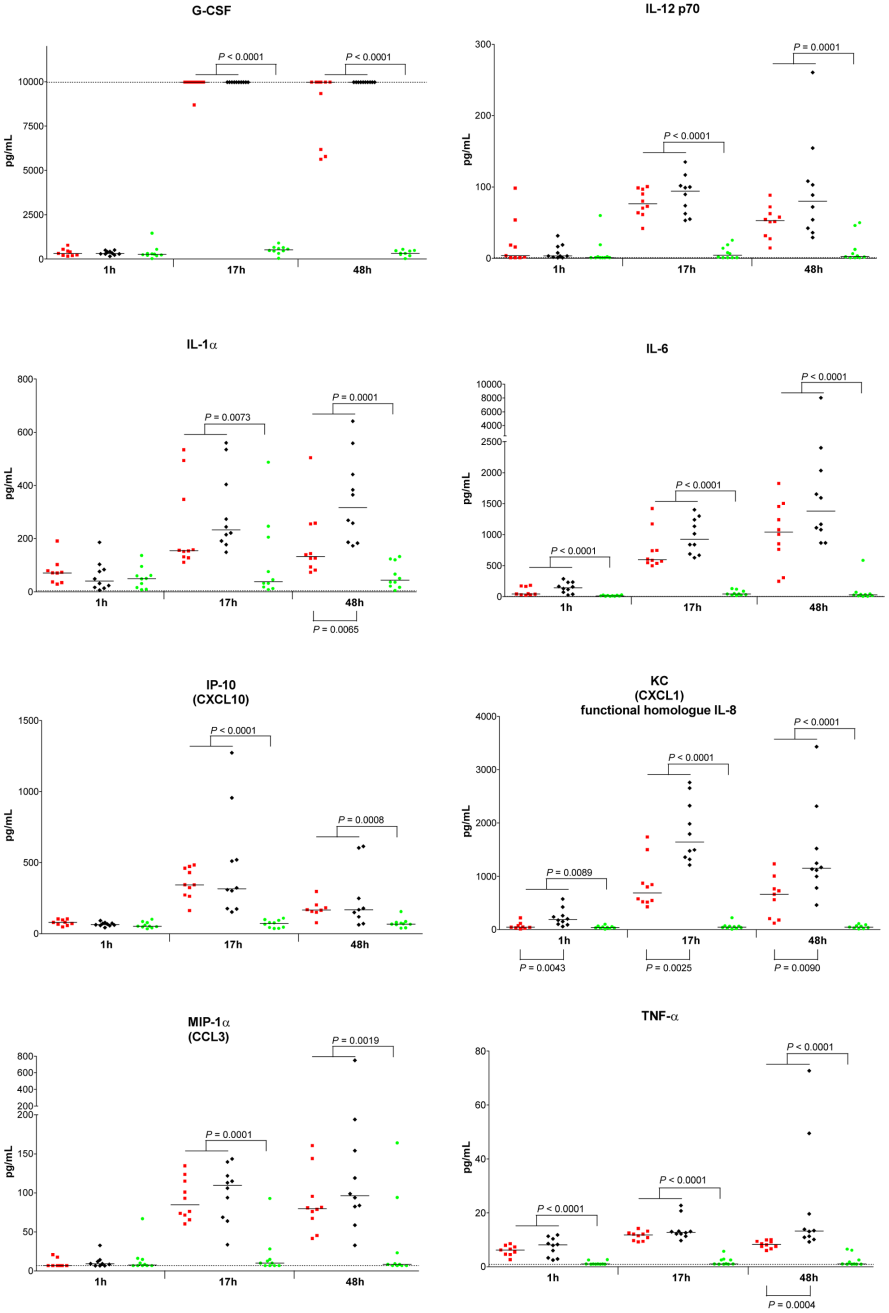
Serum cytokine levels elevated due to *S. aureus* bacteraemia in mice at indicated time points. Mice were inoculated intravenously with *S. aureus* at the LD_50_ inoculum. Each symbol represents a single mouse. Red squares represent surviving infected mice (n = 10); black diamonds represent non-surviving infected mice (n = 10); green circles represent placebo-inoculated mice (n = 10). Median values are presented by horizontal lines. Statistically significant differences in cytokine levels between infected mice versus placebo-inoculated mice are indicated above the x-axis. Statistically significant differences in cytokine levels between surviving infected mice versus non-surviving infected mice are indicated below the x-axis. *P*-values < 0.01 (Mann-Whitney *U* test) are shown. h, hours.

Comparing infected mice to placebo-inoculated mice, 21 cytokines were elevated in infected mice (*P* < 0.01; Mann-Whitney *U* test). From these, 3 cytokines (TNF-α, IL-6, and KC) were already elevated early at 1 hour after infection and remained elevated at 17 and 48 hours after infection. IL-10 was elevated at 1 and 17 hours as well, but was not elevated anymore at 48 hours. At both 17 and 48 hours after infection, 12 cytokines were elevated (G-CSF, M-CSF, IFN-γ, IL-1α, IL-1β, IL-12p70, IL-15, IL-17A, IP-10, MCP-1, MIG, and MIP-1α) in infected mice compared to placebo-inoculated mice. GM-CSF, MIP-1α, MIP-2, and RANTES were only elevated at 17 hours, while eotaxin was only elevated at 48 hours after infection. Logistic regression analysis showed that G-CSF, TNF-α, IL-1α, IL-6, IL-12p70, IP-10, KC, and MIP-1α were discriminative between infected mice and placebo-inoculated mice. Levels of these 8 cytokines in individual mice are shown in Fig. 6. Levels of G-CSF showed an impressive increase between 1 and 17 hours after infection, and remained high at 48 hours. Levels of IL-12p70, IL-1α, IL-6, KC, and MIP-1α showed an increase between 1 and 17 hours as well, although this increase was less pronounced than for G-CSF. The levels of these 5 cytokines remained elevated at 48 hours. IP-10 levels showed an initial increase between 1 and 17 hours as well, but decreased again between 17 and 48 hours. Levels of TNF-α were elevated at 1, 17, and 48 hours. The elevated cytokine levels in infected mice were not the result of animal handling during blood drawing of blood, as cytokine levels in placebo-inoculated mice were consistently low. Cytokine levels in surviving infected mice decreased over time, and were near or comparable to those in placebo-inoculated mice 2 weeks after inoculation (data not shown).

Comparing non-surviving infected mice to surviving infected mice, we also found differences in the cytokine levels. Serum levels of 5 cytokines were elevated in non-surviving infected mice compared to surviving infected mice (*P* < 0.01; Mann-Whitney *U* test). From these, KC was already elevated early at 1 hour after infection in non-surviving infected mice, and remained elevated at 17 and 48 hours after infection. The other 4 cytokines (TNF-α, IL-1α, IL-1β, and eotaxin) were elevated in non-surviving infected mice only at 48 hours after infection. Logistic regression analysis showed that TNF-α, IL-1α, and KC were discriminative between non-surviving infected mice and surviving infected mice. Each of the three cytokines showed an area under ROC curve > 0.8. Combining these cytokines in the analysis did not further improve the area under ROC curve.

## Discussion

Early diagnosis of *S. aureus* bacteremia is crucial to reduce mortality rates. In addition, optimization of antibiotic use is of high importance. To this aim, novel and reliable markers as predictors of infection outcome are urgently needed. In this study in mice, we determined whether blood culture, CRP, and cytokines are potential biomarkers for presence and outcome of *S. aureus* bacteremia. We have established a model of *S. aureus* bacteremia in mice that were *S. aureus*-free before infection. In this way, all (immune) responses in these animals were due to the inoculated *S. aureus* strain only. A clinical *S. aureus* isolate recovered from a septic patient was used at an inoculum resulting in 50% mortality of mice. This experimental design allowed comparison of surviving and non-surviving mice infected with the same *S. aureus* inoculum. Staphylococci mainly migrated to the kidneys, resulting in an increase of bacterial load and bacterial microabscesses in the kidneys were observed. Similar observations were described by Cheng *et al*. [36]. The model proved to be very reproducible, and provides a robust experimental system to investigate *S. aureus* bacteremia.

When a patient is suspected of bacteremia, blood is drawn for identification of the causative infectious agent, and cultured in broth to make sure that low bacterial blood counts will also be detected. Using this method, a quantitative blood culture cannot be assessed. In the current study in mice, we investigated whether presence of *S. aureus* bacteremia or *S. aureus* quantitative blood counts are biomarkers predicting fatal outcome of *S. aureus* bacteremia. A positive *S. aureus* blood culture did not discriminate between eventually non-surviving infected mice and eventually surviving infected mice. The *S. aureus* blood counts tended to be higher in non-surviving infected mice compared to surviving infected mice, but this was not statistically significant. We concluded that neither the positive *S. aureus* blood culture nor the *S. aureus* counts in blood could be used as biomarkers indicative for fatal infection outcome.

We included CRP as a potential biomarker for presence and fatal outcome of *S. aureus* bacteremia as this marker is frequently used in clinical practice to determine and monitor inflammation and infection in general. Circulating CRP levels directly reflect the intensity of the pathological processes that stimulate the CRP production [7]. The present study showed that CRP can be used as a biomarker for presence of severe *S. aureus* bacteremia in mice. However, serum CRP levels were not discriminative between surviving infected mice and non-surviving infected mice, and therefore CRP is not a biomarker for fatal outcome of infection. These data in mice are in concordance with findings in bacteremic patients, as CRP is a biomarker for presence of *S. aureus* bacteremia, but does not predict outcome of infection [6]. As CRP is not specific for *S. aureus* infection, elevated CRP levels will not indicate bacteremia caused by *S. aureus* only.

In addition, we assessed whether cytokines can be used as biomarker in *S. aureus* bacteremia. According to the binary logistic regression analysis, TNF-α, IL-1α, KC, G-CSF, IL-6, IL-12p70, IP-10, and MIP-1α are indicative for presence of *S. aureus* bacteremia in mice, while TNF-α, IL-1α, and KC were also potential biomarkers for fatal outcome of this infection. As the last three cytokines each had a very high area under ROC curve, and combination of these cytokines did not improve this, each of these can be used individually as biomarkers for fatal outcome. Diagnosis of *S. aureus* bacteremia cannot be based solely on elevated levels of these cytokines, as cytokine levels will rise in other infections as well. Until now, cytokine levels in mice with *S. aureus* bacteremia are not well studied. Ashare *et al.* assessed cytokine levels in a murine model simulating polymicrobial sepsis and showed that levels of TNF-α and IL-1β in liver were elevated in septic mice compared to controls [37]. Osuchowski *et al.*, using the same murine model, showed that plasma levels of MCP-1, MIP-2, and TNF-α had a robust correlation with outcome of infection [38]. Discrepancies between these studies and the present study could be explained by differences in the biomaterial in which cytokines were measured, differences in the causative infectious organism(s), and differences in cytokines included for assessment in these studies. In the present study, we selected a broad panel containing 31 cytokines, covering the main cytokines found in related studies, and including both pro- and anti-inflammatory cytokines. Most other studies measured only a limited number of cytokines, up to 16.

In the present study, 3 cytokines (TNF-α, IL-1α, and KC) emerged as biomarkers for fatal outcome of *S. aureus* bacteremia. TNF-α is primarily produced by activated macrophages and stimulates the acute phase reaction. It induces apoptotic cell death, attracts neutrophils and stimulates phagocytosis. IL-1α is also mainly produced by activated macrophages early after onset of infection, and plays one of the central roles in the regulation of immune responses. It activates lymphocyte proliferation and increases number of blood neutrophils. KC is the murine functional homologue of human IL-8 [35] and is an important neutrophil chemoattractant. Neutrophils appear to be important in *S. aureus* sepsis, as TNF-α, IL-1α and KC/IL-8 all attract these cells. In patients with *S. aureus* bacteremia, neutrophils are activated as well [39]. At both 1 and 17 and 48 hours after infection, KC was elevated in non-surviving infected mice compared to surviving infected mice. A biomarker elevated irrespective the progression of infection, such as KC, is very interesting for clinical application in view of the heterogeneity of patients experiencing bacteremia in this respect.

In the present study, we exhaustively examined blood culture, CRP and cytokines as biomarkers for presence of severe *S. aureus* bacteraemia and fatal outcome of infection in mice. This study provides evidence that in severe *S. aureus* bacteremia in mice, TNF-α, IL-1α, and KC each can be useful as biomarkers predicting fatal outcome of infection. Blood cultures (qualitative or quantitative) and CRP are not discriminative in this respect. As elevated levels of these cytokines are indicative for *S. aureus* bacteremia, these cytokines cannot be used to diagnose *S. aureus* bacteremia; instead these cytokines might be useful as biomarkers for outcome of infection in patients already diagnosed with *S. aureus* bacteremia. Several clinical studies have shown that it is difficult to evaluate changes in cytokine profile during bacteremia in patients, and this is mainly due to differences in causative infectious agents. Studies focused on a single, known causative infectious organism might be more informative. In the clinical setting, infecting *S. aureus* strains will vary between patients. In the present study in mice, only one *S. aureus* clinical isolate was used. So, we should be careful in extrapolating the results obtained in mice to the clinical setting. As the host as well as the *S. aureus* strain are both important determinants in the infectious process, and only one mouse strain and one *S. aureus* strain were included in the present study, it is currently unknown whether the observations obtained can be generalized. Therefore, we conclude that the current results in mice suggest that cytokines might be reliable biomarkers for fatal outcome of *S. aureus* bacteremia in patients. Currently, data on appropriate biomarkers are lacking and only when bacteremia is strongly suspected in patients, antibiotic treatment is started. Based on the present study in mice, a clinical study on the role of selected cytokines in predicting the infection outcome is warranted. These data will improve decisions on the start and choice of antibiotic therapy.
